# Differentiation and Regulation of Bovine Th2 Cells In Vitro

**DOI:** 10.3390/cells13090738

**Published:** 2024-04-24

**Authors:** Anmol Kandel, Lei Li, Yan Wang, Wenbin Tuo, Zhengguo Xiao

**Affiliations:** 1 Department of Animal and Avian Sciences, University of Maryland, College Park, MD 20742, USA; akandel1@umd.edu (A.K.); lixxx242@umd.edu (L.L.); 2 Mass Spectrometry Facility, National Institute of Dental and Craniofacial Research, National Institutes of Health, Bethesda, MD 20892, USA; 3 Animal Parasitic Diseases Laboratory, U.S. Department of Agriculture, Agricultural Research Service, Beltsville, MD 20705, USA; wenbin.tuo@usda.gov

**Keywords:** bovine, Th2 differentiation, IL4, IFNγ, proteomics

## Abstract

Bovine Th2 cells have usually been characterized by IL4 mRNA expression, but it is unclear whether their IL4 protein expression corresponds to transcription. We found that grass-fed healthy beef cattle, which had been regularly exposed to parasites on the grass, had a low frequency of IL4+ Th2 cells during flow cytometry, similar to animals grown in feedlots. To assess the distribution of IL4+ CD4+ T cells across tissues, samples from the blood, spleen, abomasal (draining), and inguinal lymph nodes were examined, which revealed limited IL4 protein detection in the CD4+ T cells across the examined tissues. To determine if bovine CD4+ T cells may develop into Th2 cells, naïve cells were stimulated with anti-bovine CD3 under a Th2 differentiation kit in vitro. The cells produced primarily IFNγ proteins, with only a small fraction (<10%) co-expressing IL4 proteins. Quantitative PCR confirmed elevated IFNγ transcription but no significant change in IL4 transcription. Surprisingly, GATA3, the master regulator of IL4, was highest in naïve CD4+ T cells but was considerably reduced following differentiation. To determine if the differentiated cells were true Th2 cells, an unbiased proteomic assay was carried out. The assay identified 4212 proteins, 422 of which were differently expressed compared to those in naïve cells. Based on these differential proteins, Th2-related upstream components were predicted, including CD3, CD28, IL4, and IL33, demonstrating typical Th2 differentiation. To boost IL4 expression, T cell receptor (TCR) stimulation strength was reduced by lowering anti-CD3 concentrations. Consequently, weak TCR stimulation essentially abolished Th2 expansion and survival. In addition, extra recombinant bovine IL4 (rbIL4) was added during Th2 differentiation, but, despite enhanced expansion, the IL4 level remained unaltered. These findings suggest that, while bovine CD4+ T cells can respond to Th2 differentiation stimuli, the bovine IL4 pathway is not regulated in the same way as in mice and humans. Furthermore, *Ostertagia ostertagi* (OO) extract, a gastrointestinal nematode in cattle, inhibited signaling via CD3, CD28, IL4, and TLRs/MYD88, indicating that external pathogens can influence bovine Th2 differentiation. In conclusion, though bovine CD4+ T cells can respond to IL4-driven differentiation, IL4 expression is not a defining feature of differentiated bovine Th2 cells.

## 1. Introduction

Although vaccination is the most cost-effective strategy for mitigating infectious diseases in animals, existing vaccines against most extracellular pathogens like gastrointestinal nematodes offer only partial protection upon parasite re-challenge in cattle [1,2,3,4]. Th2 cells are critical in controlling extracellular pathogens [5,6,7]; however, it is unclear if and how naïve bovine CD4+ T cells differentiate into Th2 cells [8,9]. While IFNγ-producing Th1 cells which defend the organism against invading intracellular pathogens have been commonly detected in infected or vaccinated cattle [10,11,12,13,14,15], whether IL4 protein-producing Th2 cells differentiate in cattle remains mostly unclear. Increased IL4 mRNA expression is considered a hallmark for bovine Th2 responses [16,17,18,19,20].

In mice and humans, IL4 decreases the production of IFNγ, a signature Th1 protein. Additionally, the master regulator of IL4, GATA3, indirectly cross-regulates the transcription of T-bet, the master regulator of IFNγ [21,22,23,24], promoting the development of IFNγ-negative Th2 cells which produce high levels of IL4. In contrast to data from mice and humans [25,26,27,28], antigen-specific bovine CD4+ T cell clones significantly (60–90%) co-express IFNγ and IL4 transcripts [8,9], suggesting their predominant differentiation into double-positive (IFNγ+ IL4+) Th0 cells capable of co-producing both Th1- and Th2-associated hallmark proteins. Nevertheless, despite of the controversy, an elevated ratio of IL4/IFNγ mRNA detection has been considered a distinguishing feature of bovine Th2 cells [8,29,30].

Exposure to antigens derived from extracellular pathogens typically leads to an increased frequency of IL4+ Th2 cells in the blood and lymphoid tissues of mice and humans [31,32,33,34,35]. However, in a recent study of healthy pasture-raised beef cattle [36], which had been frequently exposed to extracellular parasites such as Ostertagia ostertagi [37], we discovered a small percentage of IL4 protein-producing CD4+ cells in the blood, which was not significantly different from that of grain-fed cattle, which were less likely to be exposed to these environmental extracellular parasites [36]. These observations led us to question whether IL4+ IFNγ- CD4+ T cells are differentially distributed across other tissues such as the spleen and draining lymph nodes in pasture-raised cattle. However, our examination consistently revealed that bovine CD4+ T cells produced a limited amount of IL4 protein irrespective of the examined tissues. Thus, we investigated how naïve bovine CD4+ T cells differentiate into a typical Th2 condition in vitro, created by adopting the standard practice from mice and humans [38,39,40]. The differentiated cells were analyzed through an unbiased proteomic assay, flow cytometry, and quantitative PCR. Also, the regulation of bovine Th2 cell differentiation was tested under distinct culture conditions such as reduced TCR stimulation strength, exposure to additional recombinant bovine IL4 (rbIL4), and the presence of Ostertagia ostertagi adult worm extract in vitro.

Our results suggest that bovine CD4+ T cells differentiate into dominantly IFNγ-producing IL4-negative cells, along with a small fraction (<10%) of IFNγ- and IL4-co-expressing hybrid Th0 cells, which mostly reflects observations from the blood and lymphoid tissues of pasture-raised cattle which are routinely exposed to extracellular parasites, including *Ostertagia ostertagi*. Based on the differential regulation of proteins relative to the naïve cells, a proteomic analysis verified bovine Th2 differentiation; however, multiple pieces of evidence contrasted the notion of IL4 expression being the signature feature of bovine Th2 cells. Therefore, the expression of IL4 may not be the only signature cytokine for Th2 in cattle.

## 2. Materials and Methods

### 2.1. Cattle

The Wye Angus herd has been a closed herd since 1958. It is maintained by the Wye Research and Education Center at the University of Maryland Experimental Station in Queenstown, Maryland [41]. The steers are grazed on a pasture composed of clover and orchard grass and are supplemented with bailage and alfalfa during the winter season [42]. When the animals are 5 or 6 months old, they are weaned and split into grass-fed or feedlot (grain-fed) groups. While the grain-fed steers are processed at 14 months, the grass-fed steers are processed at 20 months [37,43]; in the case of our study, this took place at a commercial plant run by George G Ruppersberger & Sons in Baltimore, MD, USA. The Animal Care and Use Protocols in our study were approved by the UMD (R-FEB-18-06 and R-JAN-21-02) Institutional Animal Care and Use Committee. All the methods were performed in accordance with the relevant guidelines and regulations.

### 2.2. CD4+ T Cell Isolation

Blood was collected from the jugular veins of cattle at the Wye Angus Farm (UMD, Queenstown, MD, USA). PBMCs were extracted from anticoagulated blood [37,43]. The resultant single-cell suspension was incubated with FITC-conjugated anti-bovine CD4 (Clone #CC8, BioRad, Hercules, CA, USA) and PE-conjugated anti-bovine CD25 (Clone #CC63, BioRad, Hercules, CA, USA) for 30 min at 4 °C, followed by two medium washes. The final suspension included 2 × 10^7^ cells per mL. The purity of the sorted CD4+ T cells was confirmed to be greater than 92% using a FACSAria II sorter (BD, San Jose, CA, USA). These were subsequently stained with anti-bovine CD25 (Clone #LCTB2A, Washington State University (WSU), Seattle, WA, USA) to confirm CD25-negative status using a FACSCanto I (BD, San Jose, CA, USA).

### 2.3. Differentiation of CD4+ T Cells

The culture method in our study was similar to that described previously [44,45]. Briefly, anti-bovine CD3 (Clone #MM1A, WSU, Pullman, WA, USA) was added to 24-well plates at 10 µg/mL in 250 µL 1X PBS (Hyclone, Logan, UT, USA), and the coated wells are covered with replacing RP-10 medium at 4 °C overnight, which was discarded before seeding the cells. Sorted CD4+ T cells were resuspended either in freshly made Th1 or Th2 differentiation medium at a desired density, and a 1.5 mL cell suspension containing 2 × 10^5^ CD4+ T cells was seeded in the anti-bovine CD3-coated wells. The Th1 differentiation medium was made by mixing RPMI 1640, 5% FBS, L-Glutamine (200 mM), Penicillin/streptomycin (100×), and reagents in the Cell X Vivo Human Th1 cell differentiation kit (R & D system, catalogue# CDK001). The Th2 differentiation medium was prepared by mixing X-VIVO^TM^15 serum-free Hematopoietic cell medium (Lonza, catalogue # 04-418Q), Penicillin/streptomycin (100×), and reagents present in the human Th2 kit (R & D system, catalogue# CDK002). The seeded plates were incubated at 37 °C with 5% CO_2_ for 13 days for Th2 and 5 days for Th1 differentiation. Depending on cellular growth, around 300 uL of differentiation medium was replaced every 3–4 days.

### 2.4. Flow Cytometry Analysis

Th1 and Th2 cells were collected for staining on days 5 and 13, respectively. The harvested cells were aliquoted for surface and intracellular staining. For surface labeling, the cells were stained with primary antibodies for 30 min at 4 °C before being washed with 2% staining buffer (SB) to eliminate any unbound antibodies. Staining with a secondary antibody was performed in the same way. After the final wash, the cells were incubated with a fix solution for 15 min at 4 °C. Followed by one more round of SB wash, the cells were resuspended in 100 μL SB and analyzed with the FACSCalibur™ flow cytometer (BD Biosciences, Becton, NJ, USA).

Before performing intracellular staining, the cells were stimulated with a cell activation cocktail (Bio-techne, Minneapolis, MN, USA) at 37 °C with 5% CO_2_ for 4 h. The cocktail contained monensin sodium salt (1.5 mM), phorbol 12-myristate 13-acetate (0.0405 mM), and ionomycin calcium salt (0.67 mM) [46,47]. The cells were then permeabilized with a Perm/Wash buffer for 15 min at 4 °C and subsequently incubated with primary (Table 1) followed by secondary antibodies (Table 2) for 30 min at 4 °C. The isotype controls were stained using isotype antibodies, and an unstained control was also included. Finally, the cells were resuspended in 100 μL SB, and then flow cytometry was performed, acquiring a minimum of 20,000 events. Data analysis was conducted using FlowJo version 10 (Tree Star, Ashland, OR, USA).

### 2.5. Proteomics

For the proteomic analysis, 50 µg of each protein sample was separately digested with trypsin using the EasyPep mini digestion kit (Thermo Scientific, Waltham, MA, USA). Each sample was then labeled with a different TMTPro (Thermo Scientific, Waltham, MA, USA) reagent following the manufacturer’s protocol. The labeled peptides were then mixed, and the mixture was dried under vacuum. The mixed peptide sample was then dissolved in 300 µL 0.1% TFA and fractionated into 10 fractions using the high pH fractionation kit (Thermo Scientific, Waltham, MA, USA). Each fraction was dried under vacuum and reconstituted in 0.1% TFA.

A Dionex U3000 nanoHPLC system interfaced to a Thermo Scientific orbitrap Fusion Lumos mass spectrometer was used for the LC-MS/MS analysis. Around 1 µg of each fraction was injected into an Acclaim^TM^ PepMap^TM^ 100 trap column (75 µm × 2 cm, Thermo Scientific) and desalted at 5 µL/min with 100% Solvent A (0.1% formic acid in water) for 5 min. The peptides were then separated using an Acclaim PepMap^TM^ 100 nano column (3 µm, 75 µm × 250 mm) over 160 min with a linear gradient of 5–50% solvent B (80% ACN, 0.1% formic acid). The Orbitrap mass analyzer was used to detect precursor (R = 120,000) and fragment (R = 50,000) masses. Data-dependent MSMS was performed at a cycle time of 2 s. Dynamic exclusion was set to 20 s within a 10 ppm error. The collision energy was 37%, which is higher than normal to assure the efficient fragmentation of TMTPro-labeled peptides.

The Proteome Discoverer software (v. 2.5, Thermo Scientific) was used for protein identification and quantification, peptide matching and protein inference were carried out using the Sequest HT search engine against a bovine protein database downloaded from Uniprot (uniprot.org, 6 March 2020). M oxidation (+15.995) and protein N-terminal acetylation were designated as the variable modifications, while carbamidomethylating of C (+57.021), TMTPro labeling (+304.207) of K, and peptide N-Terminal were designated as the fixed modifications. Mass tolerance was 10 ppm for the precursor and 0.02 Da for the fragment masses. Percolator was used to control the false discovery rate to <1%. TMT reporter ions were quantified based on signal to noise when available, and, when the data did not have signal to noise, reporter ion intensity was used. Protein abundances were normalized to the abundance of GAPDH. A nested study design was applied for relative peptide and protein quantification. The peptide and protein ratios were first calculated within cells derived from the same cattle, then a background-based T-test was then applied to evaluate significance. Peptides with >35% coisolation or < 2 reporter ions were not included in this quantification. Principle component analysis (PCA) and the heatmap of proteins related to immune response (Gene Ontology) were generated from Proteome Discoverer.

Proteomic data are available as the MassIVE dataset MSV000091527.

### 2.6. Quantitative PCR

Cells were collected and washed with 1X PBS, and the pellets were lysed with RLT lysis buffer (Qiagen, Germantown, MD, USA). The RNeasy Micro kit (Qiagen, Hilden, Germany) was used to extract the total RNA, with the DNase treatment included in the procedure. A NanoDrop 1000 spectrophotometer was used to test RNA quality, and 400 ng of total RNA was used to synthesize first-strand cDNA using the Thermo Maxima First Strand cDNA synthesis kit (Fisher, Waltham, MA, USA) in an Eppendorf Master Gradient Cycler. A total of 400 ng of synthesized cDNA was divided into 5 ng/µL concentrations. Quantitative PCR (qPCR) amplification was performed in a C1000 Touch Thermal Cycler (BioRad, Hercules, CA, USA) using 2 µL of diluted cDNA and the IQ SYBR Green Supermix. In each reaction, 2 µL of the cDNA template and two primers (final concentration 400 nM) were used, followed by a two-step technique of denaturation at 95 °C for 3 min, 40 cycles of 10 sec at 95 °C, and an extension at 55 °C for 30 s. Melting curves were obtained after 40 cycles, and all the amplified products were validated via gel electrophoresis and sequencing.

### 2.7. Statistical Analysis

The statistical analyses were performed using Prism 8 (GraphPad Software, Inc., La Jolla, CA, USA); details can be found in the figure legends. All the data were analyzed using a one-way ANOVA with Tukey’s multiple comparison test. The asterisks denote statistical significance. * *p*  <  0.05; ** *p*  <  0.01; *** *p*  <  0.001 and **** *p*  <  0.0001. NS: not significant.

## 3. Results

### 3.1. Bovine Memory CD4+ T Cells Are a Mixture of IFNγ-Dominant Th0 Cells

**Figure 1:**
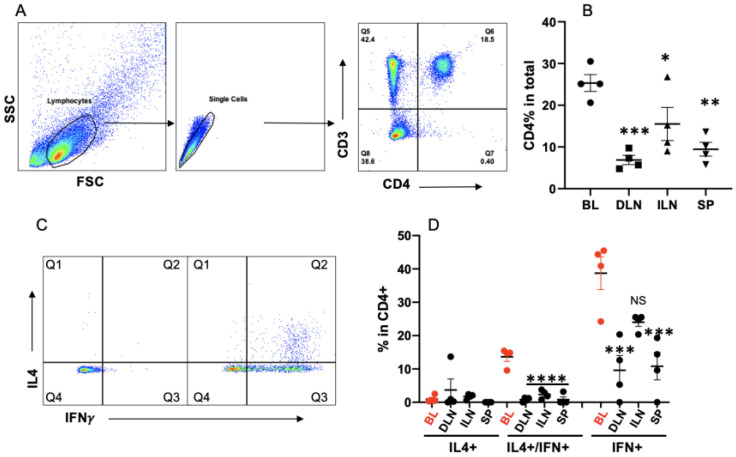
Bovine memory CD4+ T cells in healthy beef cattle are a mixture of Th1 and Th0. Lymphocytes are gated in peripheral blood mononuclear cells (PBMCs) or lymphoid tissues and examined for the expression of surface markers and cytokines. (**A**,**B**) Gating (**B**) and comparison of CD4+ T cells % in different tissues of grass-fed cattle. (**C**,**D**) Purified PBMCs or single-cell suspension of lymphoid tissue are stimulated with the activation cocktail for 4 h before intracellular staining for IFNγ and IL4, plus surface staining with antibodies to identify CD4+ T cells [36]. (**C**) Gating strategies for IFNγ- or IL4-producing cell in total CD4+ T cells. (**D**) Comparison of IFNγ or IL4 expression in CD4+ T cells. Percentage of IL4+ only: Q1; IL4+/IFNγ+ (double-positive): Q2; IFNγ+ only: Q3. Background in the isotype control is subtracted from all the data in (**D**). BL: blood; DLNs: abomasal draining lymph nodes; ILNs: inguinal lymph nodes; and SP: spleen. Data have been analyzed by one-way ANOVA with Tukey’s multiple comparison test. Asterisks indicate statistical significance compared to BL in (**B**,**D**). * *p*  <  0.05; ** *p*  <  0.01; *** *p*  <  0.001; and **** *p*  <  0.0001. NS: not significant. The same analysis has been performed in the rest of the figures, unless mentioned otherwise.

**Figure 2:**
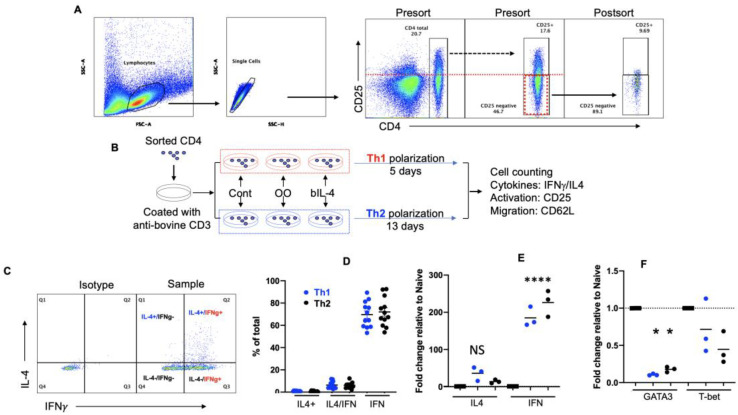
In vitro programming under Th1 or Th2 leads to Th0. (**A**) Purified PBMCs stained with anti-bovine CD4 and CD25 antibodies (direct conjugates). CD4+ T cell sorting based on CD4+/CD25-, with pre- and post-sorting purity checks. Sorted cells stimulated with anti-bovine CD3 antibody in the presence or absence of OO protein extract (3 μg/mL) and incubated in human Th1 polarization medium for 5 days or Th2 polarization medium for 13 days [66,67]. (**A**) Naïve CD4 T cells gated on lymphocytes (small and large sizes), singlets, and CD25−/CD4+. (**B**) Experimental settings for the differentiation of Th1 and Th2. Cont: control. (**C**,**D**) After 5-day (Th1) or 13-day (Th2) differentiation, cells are washed and stimulated with a cell activation cocktail for 4 h [36]. (**C**) Gating for cytokine-producing CD4+ T cells after differentiation. (**D**) Comparison of cytokine-producing cells after Th1 and Th2 differentiation. (**E**,**F**) Sorted naïve CD4+ T cells or differentiated CD4+ T cells examined for the transcription of IFNγ and IL4 (**E**) and their corresponding master transcription factors T-bet and GATA3 (**F**), all relative to naïve CD4+ T cell. Each dot represents individual cattle specimens.

The detection of an established memory T cell population is the gold standard for assessing the efficacy of vaccinations [48,49,50]. Previously, we reported that beef cattle raised on pasture demonstrated a small fraction of IL4+ CD4+ T cells in the blood [36], which was not significantly different from that in grain-fed cattle. To assess if IL4+ CD4+ T cells in grass-fed cattle are distributed more in the lymph node draining abomasum, bovine CD4+ T cells from samples of blood (BL), spleen (SP), draining lymph nodes (DLNs), and inguinal lymph nodes (ILNs) were examined for the expression of surface (CD3 and CD4) and intracellular (IFNγ and IL4) molecules. The frequency of total CD4+ T cells was highest in the BL and lowest in the DLNs (Figure 1A,B). The highest percentage of IFNγ-producing CD4+ T cells was detected in the blood, which was lowest in the lymph nodes draining the abomasum (Figure 1C,D), consistent with the notion that memory T cells predominantly exist in circulation or somatic tissues, whereas most CD4+ T cells are naïve in lymphatic tissues like the lymph nodes, thus not producing much IFNγ [51,52,53]. Of all the investigated tissues, the CD4+ T cells in PBMCs had the highest proportion (~40%) of IFNγ- and/or IL4-producing cells, with IFNγ (designated as IFN) being significantly dominant (Figure 1D). A small fraction of IL4+ CD4+ T cells were similarly distributed among the blood and different lymphoid tissues of pasture-raised cattle with no significant statistical difference, consolidating the observations from grain-fed cattle reported previously [36]. Notably, the majority of IL4 expression was restricted to double-positive Th0 cells (Figure 2D) [9]. These results suggested a differentiation of CD4+ T cells into a Th1-dominant Th0 profile in cattle.

### 3.2. In Vitro Programming under Th2 Leads to IFNγ-Dominant Th0 Mixture

Polarized Th1 and Th2 cells are well-documented in mice and humans [26,54,55]. Human Th1 differentiation kits that mainly contain human IL12 (hIL12) and Th2 differentiation kits, primarily composed of human IL4 (hIL4), are commonly used for in vitro studies [56,57]. Notably, both of these human cytokines have demonstrated effective biological activities in bovine cells [58,59,60]. With this in mind, we explored the use of human kits to differentiate bovine CD4+ T cells under typical Th2 culture conditions, keeping Th1 as a control. Previously, we discovered that, unlike in mice and humans [61,62], CD45RA expression was not associated with naïve CD4+ T cells in cattle [36,63]. Therefore, we sorted out resting CD25-negative CD4+ T cells, as in Figure 2A, and stimulated them with anti-bovine CD3 under Th2 conditions using a human differentiation kit (Figure 2B). Indeed, CD4+ T cells differentiated under human Th1 conditions, hereafter referred to as Th1 cells for convenience, resulted in typical IFNγ+ effector cells, a small fraction of which also co-expressed IL4, consistent with the observations from pasture-raised cattle (Figure 2C,D) [64]. Interestingly, the majority of the cells differentiated under a Th2 culture, referred to as Th2 cells, was also not producing IL4 proteins but rather was dominantly secreting IFNγ. Though a small fraction of IL4+ cells were induced, most of them were also positive for IFNγ, suggesting that double positive (IFNγ+ IL4+) Th0 cells were the major source of IL4 (Figure 2D). Collectively, irrespective of the culture conditions, bovine CD4+ T cell differentiated mostly into IFNγ+ IL4- cells, along with a fraction (<10%) of hybrid Th0 cells. To examine the transcriptional regulation of IFNγ, IL4, and their corresponding master transcription factors T-bet and GATA3, quantitative PCR was performed using housekeeping gene GAPDH as the reference [65]. In line with protein expression data, we observed a significant (>100-fold) enhancement of IFNγ mRNA expression in both Th1 and Th2 cells compared to naïve CD4+ T cells. In contrast, IL4 mRNA was moderately enhanced (>10-fold) but with no significant statistic difference (Figure 2E). Of note, the master transcription factors, T-bet and GATA3, were highly co-expressed in the naïve CD4+ T cells, but the level of GATA3 in Th2 and that of T-bet in Th1 was significantly lower than those in naïve CD4+ T cells (Figure 2F), suggesting that the hallmark master regulators observed in mice and humans show an opposite trend in cattle. In summary, in vitro CD4+ T cell differentiation under a Th2 culture generates a Th1-dominant Th0 phenotype, consistent with the memory CD4+ T cell profile in the blood and lymphoid tissues of grass-fed beef cattle (Figure 1D) and in line with data published in our recently published papers [36,63].

### 3.3. Validation of Th2 Differentiation

To validate if the differentiated cells under Th2 conditions were actually Th2 cells, rather than focusing only on IFNγ and IL4 expression, we conducted an unbiased proteomic assay and analyzed the proteins using an ingenuity pathway analysis (IPA) software.

**Figure 3:**
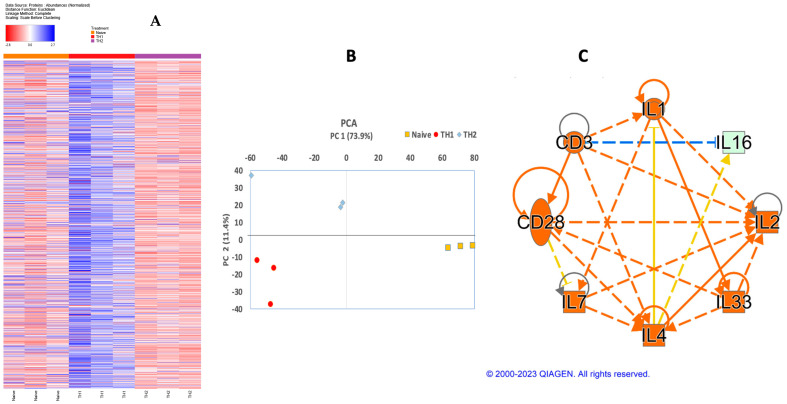
Th1/Th2 differentiations drive different effector functions. Quantitative proteomic analysis of naïve and differentiated CD4+ T cells has been carried out using LC-MS/MS following multiplexing with TMT and high pH fractionation. Heatmap of differentially expressed proteins in naïve (Nai), Th1, and Th2 cells with red indicating upregulation and blue indicating downregulation (**A**). Heatmap (after normalization to GAPDH) of identified proteins demonstrates that the cells differentiated under naïve Th1- and Th2-cultures are distinctly clustered. (**B**) Principle component analysis. (**C**) IPA pathway analysis has been performed to predict upstream cytokines and network analysis based on the predicted activation and inhibition states of the upstream regulators identified in the Th2 vs. naïve group. The data are based on the 1.5-fold up or down based on the proteins of Th2/Th1 based on the average of three biological replicates.

Multiplexing with Tandem Mass Tag (TMT) followed by high pH fractionation and LC-MS/MS led to the identification of 4212 proteins from three biological replicates in each population (Supplementary Table S1). Indeed, the Th1 and Th2 polarization regulated different groups of proteins (Figure 3A), most of which were overlapping structural proteins. Notably, Th1, Th2, and the naïve cells fell into different clusters upon principal component analysis (Figure 3B), suggesting that the cells induced under Th2 conditions demonstrated a distinct phenotype compared to naïve and Th1 cells. Considering 1.5-fold as the cutoff value (*p* < 0.05), a comparison with naïve CD4+ T cells revealed 422 differentially expressed proteins in the bovine Th2 cells and 397 in the Th1 cells (Supplementary Tables S2 and S3). Furthermore, based on the Th2 differential proteins, the ingenuity pathway analysis (IPA) predicted typical upstream regulators associated with Th2, including IL4, IL7, IL1β, and IL33 (Figure 3C), suggesting a typical Th2 differentiation. Th2 and Th1 differentiation is tightly regulated by specific master transcription factors, corresponding in a way such as T-bet for IFNγ and GATA3 for IL4 [38,68,69,70]. Indeed, based on the differentially expressed Th1 and Th2 proteins, TBX21, the gene encoding T-bet, was predicted to be activated in both Th1 and Th2 (Table 3), consistent with the enhanced IFNγ expression in Figure 2D,E. Nonetheless, based on the differentially expressed Th2 proteins, GATA3, which plays major role in maintaining IL4 expression in mice and humans, was not predicted as the upstream molecule (Table 3), consistent with reduced GATA3 transcript and unchanged IL4 mRNA/protein detection after differentiation under a typical Th2 culture (Figure 2E,F). Of note, consistent with those in the mice and humans, transcription factors like AP-1, E2F2, Maf, MYB, and NF-ϏB (Table 3) [71,72,73,74,75,76,77,78,79,80,81], as well as cytokines such as IL4, IL1, and IL33 (Figure 3C, Supplementary Table S4) [81,82,83,84,85,86,87], were predicted as upstream factors in bovine Th2 differentiation. A unbiased proteomic analysis validated general Th2 differentiation in vitro, which bears some differences compared to that in humans and mice.

### 3.4. Th2 Differentiation Is Sensitive to TCR Stimulation Strength

**Figure 4:**
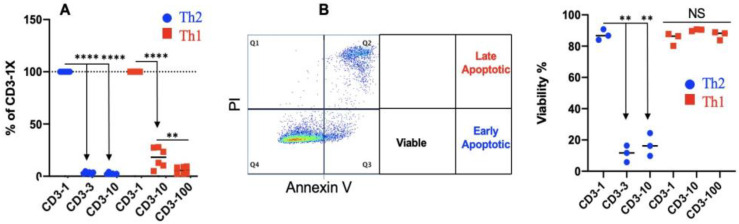
Th2 differentiations is sensitive to the strength of TCR stimulation. Naïve CD4+ T cells are differentiated under Th2 and Th1 conditions as described in Figure 2B, with a reduced CD3 concentration for coating. 1×: original concentration, 5 µg/mL; 10×: 0.5 µg/mL; and 100×: 0.05 µg/mL. Differentiated T cells are harvested for examination. (**A**) Comparison of expansion relative to 1× anti-CD3. (**B**) Comparison of viability.

In mice, reducing TCR stimulation strength allowed one to shift CD4+ differentiation toward the IL4+ phenotype [88,89,90,91,92]. To investigate if IL4 expression could be boosted in a similar way, isolated naïve CD4+ T cells were stimulated with plate-bound anti-bovine CD3 antibodies coated at varied concentrations under both Th1 and Th2 conditions, as illustrated in Figure 2B. Even three-fold dilution (3×) of anti-bovine CD3 reduced Th2 proliferation by more than 95%, while cellular expansion under Th1 conditions was reduced by 80% at a 10× dilution (Figure 4A). Furthermore, the viability of the tiny number of surviving cells in Th2 cells was reduced to roughly 20%, demonstrating that even a 3× dilution of CD3 essentially eliminated Th2 survival (>99%) compared to the 1× CD3 control (Figure 4B). In Th1 cells, the viability of the remaining cells at 10× diluted CD3 was similar to that induced in the original anti-CD3 concentration (1×), about 90% (Figure 4B), indicating that the survival of differentiating bovine Th2 cells is more dependent on intense TCR stimulation than Th1 cells. Therefore, in contrast to mice and humans, a lower TCR stimulation strength is detrimental to the survival of bovine Th2 cells.

### 3.5. Extra Recombinant Bovine IL4(rbIL4) Leads to Enhanced Th2 Differentiation

**Figure 5:**
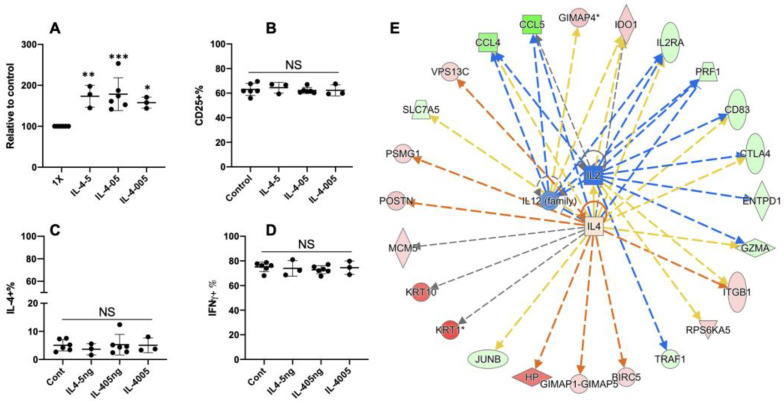
Extra recombinant bovine IL4 leads to enhanced Th2 differentiation. Naïve CD4+ T cells are differentiated under Th2 condition in the presence of additional recombinant bovine IL4. IL4 is tested at three different concentrations: 0.05 ng/mL, 0.5 ng/mL, and 5 ng/mL. Comparison of expansion (**A**), CD5 expression (**B**), and IFNγ and IL4 expression (**C**). (**D**) Th2 cells differentiated in the presence of 0.5 µg/mL recombinant bovine IL4 are analyzed for their protein profiles, in contrast to Th2 control cells. IPA is used to predict the effect function of the additional rbIL4. (**E**) The network analysis is based on the differentially expressed proteins between rbIL4+Th2 vs. Th2.

To test if the exposure to extra exogenous rbIL4 could enhance IL4 expression, purified naïve CD4+ T cells were cultured under Th2 condition for 13 days as mentioned in Figure 2B, in the presence of rbIL4 introduced at various concentrations. The cells grew robustly when rbIL4 was added at 0.05–5 ng/mL concentrations, ending up with a 2-fold increase compared to the control (Figure 5A). However, no changes were observed in CD25 (Figure 5B) or IFNγ/IL4 expression (Figure 5C,D, Supplementary Figure S1). To confirm if this enhanced proliferation was induced through IL4, 0.5 ng/mL recombinant bovine IL4 was applied to the Th2 condition, and the protein profiles of Th2 cells were contrasted with those derived from the Th2+ rbIL4 culture. There were 175 differential proteins (Supplementary Table S5), of which 20 molecules were related to the IL4-related effector pathways (Supplementary Table S6).

In mice and humans, the presence of IL4 into the Th2 culture can enhance the expression of Th2 transcription factors, STAT6 and GATA3 [21,22], which are indispensable for the establishment of an IL4-secreting positive feedback loop. Nevertheless, our analysis based on 175 differentially expressed proteins did not predict STAT6 and GATA3 as upstream factors in rbIL4-treated bovine Th2 cells, which were observations in line with the lack of IL4 upregulation in the qPCR and flow cytometry data (Figure 2D,E). While the predicted effector pathways were related to IL4, supported by the detection of 20 targeted proteins (Figure 5E, Supplementary Table S6), the activation score of IL4 was predicted to be low (0.219, Supplementary Table S7), which was because the software detected few discrepancies between the functional features of bIL4 and its murine and human counterparts (Highlighted in Supplementary Table S6). The IPA analysis suggested that rbIL4 plays a partially different role in the bovine Th2 differentiation process compared to its counterparts in mice and humans.

### 3.6. Ostertagia Ostertagi Extract (OO) Leads to the Inhibition of Th2 Differentiation

**Figure 6:**
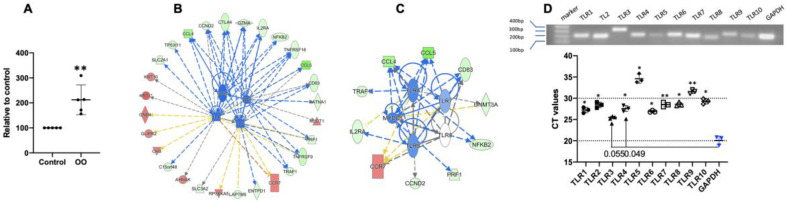
Parasite extract leads to the inhibition of Th2 differentiation. Naïve CD4 T cells are differentiated in the presence or absence of OO under Th2 conditions, as described in Figure 2B. Cells are harvested and analyzed for changes in their protein profiles, and the differentially expressed proteins are further analyzed by IPA. (**A**) Comparison of cell expansion influenced by OO. (**B**) Predicted effector function of OO on Th2 differentiation. (**C**) Predicted OO-affected network. The network analysis is based on the predicted activation and inhibition states of the upstream regulators identified in the Th2+OO versus Th2. (**D**) Detection of mRNA of TLRs. Naïve CD4 T cells are analyzed for the presence of TLRs.

Extracellular parasites regulate Th2 differentiation in mice and humans in vitro [93]. OO, which is composed of proteins extracted from the *Ostertagia ostertagi* worm, has effectively demonstrated ability to modulate neutrophil functions in cattle [94], eventually inhibiting the activation of bovine CD4+ T cells [43,65]. To test if differentiated bovine Th2 cells are sensitive to the OO extract, sorted naïve bovine CD4+ T cells were cultured under an anti-CD3-stimulated Th2 condition in the presence or absence of the OO protein extract, as indicated in Figure 2B, and the harvested cells were analyzed by flow cytometry and a proteomic analysis. To our surprise, the treatment with OO led to cellular proliferation by 2–3-fold under Th2 conditions (Figure 6A), without noticeable changes in IFNγ and IL4 production (Supplementary Figure S2). Based on the differential regulation of 83 proteins (Supplementary Table S8), OO inhibited key nodes including CD3, CD28, CD40, IL2, IL7, IL15, and NF-ϏB, which are critical regulators of Th2 cell differentiation (Figure 6B, Supplementary Table S9) [8,93]. Of note, the TLR/MYD88 pathways were also found to be inhibited, especially through TLR4, 7, and 9 (Figure 6C). Despite the fact that bovine innate immune cells express a broad range of TLRs [95], it was previously unknown if naïve bovine CD4+ T cells expressed TLRs like in mice and humans [96,97]. For the first time, we confirmed that naïve bovine CD4+ T cells express all the 10 TLRs in PCR, and their expression levels, except for that of TLR3, were abundant within 10 cycles compared with the housekeeping gene GAPDH (Figure 6D). It seems that bovine Th2 differentiation can be inhibited by extracellular pathogens through different pathways, such as suppression via TLR/MYD88, at least in vitro.

## 4. Discussion

In the blood and lymphoid tissues of pasture-raised cattle, the majority of the identified CD4+ T cells was IFNγ + IL4-, and a small fraction of double-positive Th0 cells were mostly producing IL4 protein, which was almost similarly reflected by in vitro differentiated bovine Th2 cells. The analysis of the differentially expressed proteins suggested that Th2 differentiation was not associated with IL4 expression in cattle, which remained unenhanced despite manipulating the culture conditions in manners previously published with respect to mice and humans.

Grass-fed cattle specimens, which are fed in pastures, are routinely exposed to the infective larvae of extracellular parasites such as *Ostertagia ostertagi*, but grain-fed cattle specimens raised on feedlot-based diets are not exposed to these environmental pathogens. Therefore, we speculated that the memory CD4+ T cells in the blood of grass-fed cattle would produce more IL4 proteins than those in the grain-fed group. Contradicting our hypothesis, the peripheral CD4+ T cells from the grass-fed cattle were producing a significantly low amount of IL4 compared to that of IFNγ, demonstrating a profile similar to that in the grain-fed group [8]. While mice exposed to nematodes such as *Nippostrongylus brasiliensis* and *heligmosomoides polygyrus* induce a high frequency of IL4+ Th2 cells in the spleen and mesenteric lymph nodes compared to those in the peripheral lymph nodes [31,32,33,34], we speculated whether IL4+ CD4+ T cells are distributed unevenly among different lymphoid tissues, such as the spleen (SP) and lymph node-draining abomasum (DLN), compared to those in the inguinal lymph nodes (ILNs) and the blood (BL). Once again, all these tissues reflected profiles similar to the profile detected in the blood (Figure 1D), contrasting observations drawn from mice [98,99]. As an IFNγ-dominant Th0 profile was consistently detected irrespective of exposure to different dietary and environmental conditions, we speculated that, perhaps, bovine CD4+ T cells spontaneously differentiate into a dominantly IFNγ-producing mixed profile, which could be examined under in vitro Th2 conditions, paralleling the standard protocol in mice and humans [38,39,40].

In line with observations derived from the blood and lymphoid tissues of healthy pasture-raised cattle, naïve bovine CD4+ T cells did not exclusively differentiate into IL4 protein-producing cells. Only a small fraction of differentiated cells was IL4+, which mostly consisted of double-positive Th0 cells. To further investigate if the protein expression tendency was reflected at the mRNA level, we evaluated transcriptional regulation of hallmark cytokines and their master regulators, which were in line with the earlier observations. Differently from mice and humans, in which the IL4 transcript level keeps on increasing after two days of stimulation [100], IL4 mRNA expression in differentiated bovine CD4+ T cells was not significantly enhanced compared to that in the naïve counterpart [29]. Furthermore, unlike the gradual upregulation of GATA3 mRNA following a few hours of stimulation under murine and human Th2 cultures [39,101,102,103,104,105], the GATA3 transcript was at the highest level in the naïve bovine CD4+ T cells was and downregulated significantly after differentiation (Figure 2F). While IL4 expression and IFNγ exclusion are associated with a lack of T-bet expression in GATA3-expressing Th2 cells in mice and humans [38,39,40,70,103,106,107,108,109,110], the absence of strict cross-regulations between the master regulators might be somehow related to IFNγ-dominant Th0 induction in cattle.

Since the cells differentiated under the Th2 culture were not highly producing the IL4 protein, naturally, we then questioned if in vitro differentiated cells were real Th2 cells, so we analyzed them through an unbiased proteomic assay combined with IPA analysis. Based on 422 differentially expressed proteins with respect to the naïve cells, our analysis revealed two key observations. While the predicted upstream factors were mostly consistent with Th2 differentiation in mice and humans, some cattle-specific features were noticed that partly explained why IL4 expression in these cells was low. Consistent with mice and humans, IL4, IL33, IL1, IL2, and IL7, which are key cytokines known for the differentiation, proliferation, and survival of Th2 cells [81,82,83,84,85,86,87,111,112,113,114,115,116,117,118,119,120,121,122], and transcription factors including AP-1, E2F2, Maf, MYB, Jun-B, and NF-κB, [71,72,73,74,75,76,77,78,79,80,123,124,125], which are essential for driving the Th2 transcriptional program, were similarly predicted in cattle. However, neither STAT6 nor GATA3, which are the molecules involved in chromatin remodeling and increased IL4 transcription in mice and humans [21,22], were predicted as upstream regulators in bovine Th2 differentiation, partly explaining why IL4 protein detection in the differentiated bovine Th2 was consistently low. Of note, STAT5, which has been suggested in GATA3-independent Th2 differentiation in mice [126,127], was also predicted as an upstream factor in cattle, with its z-score suggesting activation, supporting the argument that bovine Th2 differentiation was not strongly associated with IL4 mRNA/protein detection in cattle.

Although the IPA validated Th2 differentiation in cattle, IL4 expression in the differentiated cells was fairly low, which impelled us to explore strategies for optimizing IL4 expression in differentiated bovine Th2 cells. Therefore, we adopted standard practices from murine and human experiments. As weakened TCR stimulation has been correlated with IL4 enhancement under in vitro cultures [88,89,90,91], we reduced the concentration of anti-bovine CD3 used for stimulating bovine CD4+ T cells under typical Th2 conditions. Despite the fact that a weakened TCR stimulation strength did not lead to a significant alteration in IL4 protein expression, it was linked to decreased viability and diminished expansion of cells under Th2 conditions, a phenomenon not observed to the same extent in the Th1 controls. While the survival of bovine Th2 cells relied on robust TCR stimulation, their IL4 expression remained unaffected by TCR stimulation strength.

Next, we tested if exogenously supplied bIL4 could somehow overcome the limited IL4 secretion by the differentiated bovine Th2 cells. Despite sharing similar N-glycosylation sites [128,129], a high concentration of hIL4 was required for mimicking the biological effect of bIL4 on bovine cells [130], which could be due to a relatively low sensitivity and/or affinity of human analogues to the bovine receptors. To rule out this possibility, we added appropriately glycosylated rbIL4 into the Th2 culture; however, its effect on IL4 production was almost null at any added concentration, which was partially in line with previously published reports in cattle [131]. Unlike in mice and humans, where the addition of IL4 in a culture inhibits IFNγ secretion while promoting that of IL4 [100,132,133,134], neither IL4 upregulation nor IFNγ downregulation was noted in cattle, highlighting the fact that IFNγ and IL4 cross-regulation is not common in bovine species. Distinctly, bIL4 failed to induce arginase activity in macrophages in vitro [135], and its porcine analogous mediated the inhibition of B cell responses in pigs [136], so heterogeneity in the functions of this cytokine across species could be speculated.

To find out why exposure to rbIL4 did not increase IL4 expression in the differentiated cells, we conducted a further analysis in the IPA. Evidently, we found that bovine IL4 seems to have a partially different role in bovine Th2 differentiation compared to that in mice and humans. Specifically, rbIL4 addition into the Th2 culture led to the regulation of several proteins, distinctly from what occurs in mice and humans (Supplementary Table S4). Specifically, opposite to mice and humans, IL4 treatment was associated with the downregulation of the IL2 receptor (IL2R) [137,138], the downmodulation of Jun-B induction [139,140], and the suppression of CD83 (co-stimulatory molecule) expression in cattle (Supplementary Tables S4 and S5) [141,142,143,144]. Also, IL4 treatment is known to enhance the expression of the IL3 receptor (IL3R) in activated human CD4+ T cells; nevertheless, the treatment of bovine IL4 (bIL4) did not lead to the upregulation of IL3R in bovine Th2 cells [145]. Our IPA analysis highlighted that a lack of IL4 expression in bovine Th2 cells might be related to the unique biological functions of bovine IL4.

Finally, we asked the most critical question, which was whether bovine CD4+ T cell differentiation under in vitro Th2 conditions was sensitive to regulation from extracellular pathogens, which has been commonly observed in their murine and human counterparts [93,146,147,148,149]. Remarkably, the OO extract elicited enhanced the proliferation of Th2 cells by several-folds without significantly changing IFNγ and IL4 expression, which was in contrast to its inhibitory effect on the expansion of PBMCs reported before [150,151,152]; this discrepancy might be related to a difference in methodologies and culture duration. While we co-cultured CD3-stimulated sorted naïve bovine CD4+ T cells with the parasite protein extract, whole PBMCs had been used in the aforementioned experiments. Our results are partially in line with the proliferation of CD4+ T cells from healthy cattle in response to *Dictyocaulus viviparous* homogenate and *Theileria annulata*-infected cells [153,154]. Based on the differential expression of proteins, IPA strongly predicted the activation of T cell proliferation pathways in the Th2+ OO group when contrasted with Th2 cells.

The presence of the OO extract in the culture inhibited three critical signals involved in bovine Th2 cell differentiation in addition to those via the TLR/MYD88 pathways, suggesting a potential mechanism to avoid effective immunity in cattle [147,155,156,157,158]. Noticeably, CD3, which mimics antigen stimulation (first signal), CD28, which provides co-stimulation (second signal), and cytokines like IL4 and IL33, which provide polarizing third signals, were found to be inhibited in the Th2+OO culture [111,159,160]. More importantly, NF-kB signaling, which can be inhibited by extracellular pathogens such as the *Schistosoma* species in humans [93], was also found to be inhibited (Supplementary Table S7). Additionally, the ligands present in the OO extract seemed to further suppress the TLRs/MYD88 pathways, which is partially consistent with the published literature, such as the downregulation of TLR2 expression in *Filarial* and *Schistosoma japonicum* infections [161,162] and the inhibition of MYD88 signaling by the products derived from helminths [163,164]. While murine CD4+ T cells can express transcripts of TLRs from 1 to 9, which have been mostly similarly found in humans, excluding those of TLR 6 and 8 [165,166,167,168,169,170], we hereby report that naïve bovine CD4+ T cells express 10 TLRs. We believe that, at the resting stage, naïve CD4+ T cells might also perform innate immune functions. Collectively, the data indicated that OO might regulate Th2 differentiation through multiple pathways, including those via TLR/MYD88, to avoid effective Th2 responses in cattle.

One key observation consistently noticed in cattle was that a majority of IL4+ cells were hybrid Th0. In mice and humans, IFNγ and IL4 expression in CD4+ T cells is mostly mutually exclusive [25,27,28], and only a small fraction of hybrid cells co-express IFNγ and IL4. These cells were traditionally characterized as Th0 cells and, recently, have been renamed and re-characterized as Th_1+2_ cells [102,171,172,173,174,175]. Partially different from the situation in mice and humans, the evaluation of pathogen-specific CD4+ T cell clones revealed predominant (60–90%) co-expression of IFNγ and IL4 transcripts in cattle [9,20,176,177,178], which the authors designated as a bovine Th0 feature. Our data partially support the previously published data as, at the transcript level, co-expression was found to be relatively common in cattle; however, we further demonstrated that, at the protein level, only a small fraction (<10%) of cells represent actual Th0 cells. Of note, the highest levels of T-bet and GATA3 mRNA are co-expressed in naïve bovine CD4+ T cells, and, therefore, the characterization of differentiated Th0 cells based on transcript co-expression of master regulators has limitations in cattle.

While previously we have published that hIL12 can activate bovine CD4+ T cells and enhance their IFNγ expression [43,131], the lack of detection of IL12 in the Th1 proteome and that of transcription factor STAT4 as an upstream factor suggest that bovine Th1 differentiation might be very distinct in cattle [70,179,180,181]. On the other hand, while the IL4-driven Th2 kit was able to drive bovine Th2 differentiation, the process was not found to be associated with IL4 expression.

## 5. Conclusions

Our findings from the evaluation of Wye Angus cattle challenge the conventional belief that bovine Th2 cells are marked by a high IL4/IFNγ mRNA ratio. Although the naïve bovine CD4+ T cells responded to Th2-related cues, the differentiated cells dominantly produced high amounts of IFNγ proteins with a limited expression of IL4, which was supported by data from other animals. These results warrant that, though IL4 may serve as a driving force for Th2 differentiation, it may not be the primary cytokine produced by differentiated bovine Th2 cells, which underscores the need to revisit the Th1/Th2 paradigm in cattle in light of more advanced technology. While this study focused on polyclonal populations, our ongoing research aims to investigate antigen (OO)-specific CD4+ single-cell populations, a focus which will generate more definitive answers in the future.

## Figures and Tables

**Table 1:** Primary antibodies.

Specificity	Clone	Isotype	Colors	Source
bCD3	MM1A	IgG1	-	WSUMAC
bCD4	CC8	IgG2a	FITC	Bio-Rad
bCD25	LCTB2A	IgG3	PE	WSUMAC
bCD62L	BAQ92A	IgG1	FITC	WSUMAC
bIFNγ	CC302	IgG1	PE	Bio-Rad
bIL4	CC303	IgG2a	-	Bio-Rad

**Table 2:** Secondary antibodies.

Specificity	Secondary Antibodies	Source
IgG1	Anti-mouse IgG1-APC	BioLegend
IgG2a	Anti-mouse IgG2a-APC	BioLegend

**Table 3:** Predicted activation (Z score ≥ 2) of upstream transcription factors based on differentially expressed proteins in the Th1 vs. naïve and Th2 vs. naïve groups. N/A refers to not available.

Upstream Regulator		Th1/Naïve		Th2/Naïve				
	Z-Score	Predicted Activation State	*p*-Value	Target Molecules in Database	Z-Score	Predicted Activation State	*p*-Value	Target Molecules in Database
CEBPB	3.466	Activated	2.54 × 10^−12^	36	2.859	Activated	3.16 × 10^−15^	41
ETV5	2	Activated	0.0167	5	2.449	Activated	0.000158	8
MYC	4.449	Activated	4.51 × 10^−6^	40	3.667	Activated	7.34 × 10^−11^	52
TBX21	2.449	Activated	9.78 × 10^−4^	6	2	Activated	0.0308	4
FOXM1	2.47	Activated	2.3 × 10^−6^	12	N/A			
AP1	N/A				2.372	Activated	2.05 × 10^−2^	7
E2F2	N/A				2	Activated	0.00593	5
MAF	N/A				2.219	Activated	8.91 × 10^−5^	7
MYB	N/A				2.157	Activated	0.00012	11
NFKB1	N/A				2.578	Activated	6.56 × 10^−5^	15

## Data Availability

Data are contained within the article and Supplementary Materials.

## Supplementary Materials

The following supporting information can be downloaded at https://www.mdpi.com/article/10.3390/cells13090738/s1. Table S1: A list of 4212 proteins identified through LC-MS/MS in three biological replicates. Table S2: A list of 422 proteins that are differentially expressed in differentiated bovine Th2 cells, with their protein abundance ratios relative to those in the naive CD4+ T cells. Table S3: A list of 397 proteins that are differentially expressed in differentiated bovine Th1 cells, with their protein abundance ratios relative to those in naive CD4+ T cells. Table S4: Predicted activation or inhibition states of key signaling molecules obtained from the upstream analysis in the Th2 versus naïve group. Table S5: A list of 175 proteins that are differentially expressed in rbIL4+ Th2 cells, with their protein abundance ratios relative to those in naive CD4+ T cells. Table S6: A list of 20 target molecules detected in the dataset related to the IL4-related effector pathways. Table S7: Predicted activation states of major cytokines differentially expressed in the rbIL4+Th2 versus Th2 group. Table S8: A list of 83 proteins that are differentially expressed in differentiated bovine Th2 cells in the presence of OO relative to those in the Th2 only control. Table S9: Predicted inhibition states of molecules obtained from the upstream analysis of Th2+OO versus Th2 group. Figure S1. Extra recombinant bovine IL4 leads to enhanced Th2 differentiation. Figure S2. OO parasite extract leads to inhibition of Th2 differentiations.
