# Supplementary material for: Differentiation and Regulation of Bovine Th2 Cells In Vitro
Source: Cells. 2024 Apr 24;13(9):738. doi: 10.3390/cells13090738 (PMC11083891; doi:10.3390/cells13090738)
Supplement: Supplementary file 1 [file cells-13-00738-s001.zip › Supplementary figures_20240425.pdf]

Article

Differentiation and Regulation of Bovine Th2 Cells in vitro

Anmol Kandel[1], Lei Li[1**], Yan Wang[2*], Wenbin Tuo[3] and Zhengguo Xiao[1*]**

[1] Department of Animal and Avian Sciences, University of Maryland, College Park, 20742, MD, USA; akandel1@umd.edu (A.K.); lixxx242@umd.edu (L.L.)
[2] Mass Spectrometry Facility, National Institute of Dental and Craniofacial Research, National Institutes of Health. Bethesda, MD 20892-4370, USA; yan.wang2@nih.gov
[3] Animal Parasitic Diseases Laboratory, USDA/ARS, Beltsville, MD 20705 USA; wenbin.tuo@usda.gov
* Correspondence: xiao0028@umd.edu; Tel.: +1-301-405-6258; yan.wang2@nih.gov; Tel.: +1-301-347-8027.

Figure S1.

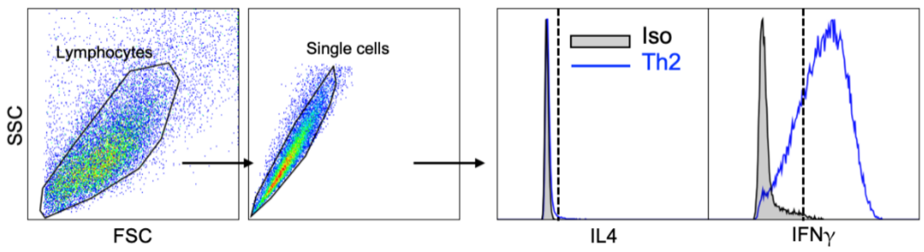

Figure S1. Extra recombinant bovine IL4 leads to enhanced Th2 differentiation. Naïve CD4+ T cells were differentiated under Th2 condition in the presence of additional recombinant bovine IL4. Gating strategy for IFNγ and IL4.

Figure S2.

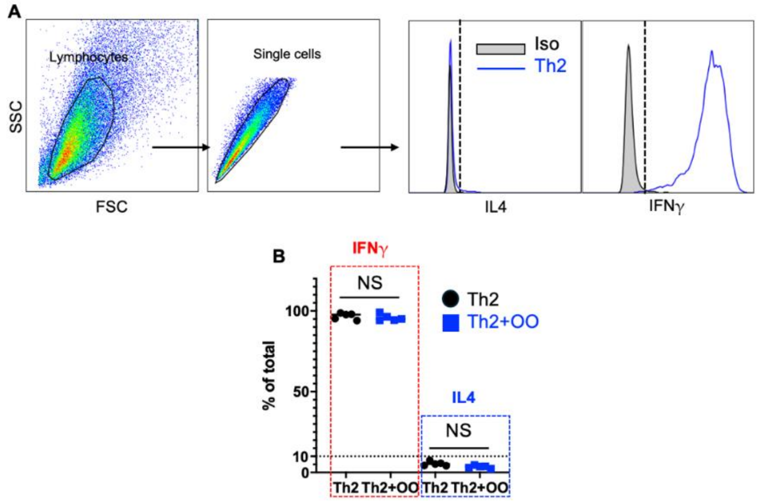

Figure S2. OO parasite extract leads to inhibition of Th2 differentiations. Naïve CD4 T cells were differentiated in the presence or absence of OO under Th2 condition as described in **Fig 2B**. Cells were harvested and analyzed for production of IFNγ and IL4. A: Gating strategy for IFNγ and IL4. B: Comparison of production of IFNγ and IL4 in Th2 vs Th2+OO.
